# Renal cell carcinoma-derived exosomes deliver lncARSR to induce macrophage polarization and promote tumor progression via STAT3 pathway

**DOI:** 10.7150/ijbs.70289

**Published:** 2022-05-01

**Authors:** Wei Zhang, Xiaoxiao Zheng, Yisong Yu, Li Zheng, Jiahua Lan, Ying Wu, Hao Liu, An Zhao, Hang Huang, Wei Chen

**Affiliations:** 1 Department of Urology, Tongde Hospital of Zhejiang Province, Hangzhou, Zhejiang 310012, China.; 2 Cancer Institute of Integrated Traditional Chinese and Western Medicine, Key laboratory of cancer prevention and therapy combining traditional Chinese and Western Medicine of Zhejiang Province, Zhejiang Academy of Traditional Chinese Medicine, Tongde Hospital of Zhejiang Province, Hangzhou, Zhejiang 310012, China.; 3 College of Life Sciences, Zhejiang Chinese Medical University, Hangzhou, 310053, China.; 4 Department of Tumor Research Institute, Cancer Hospital of the University of Chinese Academy of Sciences, Zhejiang Cancer Hospital, Hangzhou, China.; 5 Department of Urology, The First Affiliated Hospital of Wenzhou Medical University, Wenzhou 325000, China.

**Keywords:** Tumor-derived exosomes, Renal cell carcinoma (RCC), Macrophage polarization, lncARSR, STAT3

## Abstract

Tumor-derived exosomes play a pivotal role in regulating tumor progression by mediating crosstalk between tumor cells and immune cells such as macrophages within the tumor microenvironment. Macrophages can adopt two distinct polarization statuses and switch between M1 or M2 activation phenotypes in response to the different external stimuli. However, the role of tumor derived exosomes in the macrophage phenotypic switch and tumor development have not been elucidated in renal cell carcinoma (RCC). Here we found that high macrophage infiltration was associated with worse prognosis in RCC patients, therefore we propose our hypothesis that RCC derived exosomes might directly influence macrophage polarization and thus promote tumor progression. Both cell-based *in vitro* models and orthotopic transplantation *in vivo* tumor models were constructed and ELISA, flow cytometry, and macrophage functional studies were performed to investigate whether and how RCC-derived exosomes regulate macrophage polarization and tumor growth. The results found that these exosomes promote macrophage polarization, cytokine release, phagocytosis, angiogenesis, and tumor development. Further study revealed high amount of a recently discovered lncRNA called lncARSR in RCC-derived exosomes. Overexpression of lncARSR induced phenotypic and functional changes of macrophages *in vitro* and promoted tumor growth *in vivo*, while knockdown of lncARSR by siRNA disrupted the exosomes-mediated macrophage polarization. LncARSR interacts directly with miR-34/miR- 449 to increase STAT3 expression and mediate macrophage polarization in RCC cells. Together, RCC-derived exosomes facilitate the development of tumor through inducing macrophage polarization via transferring lncARSR, suggesting that RCC-derived exosomes, lncARSR and STAT3 are the potential therapeutic targets for treatment of RCC.

## Introduction

Renal cell carcinoma (RCC) is a malignant and heterogeneous cancer derived from renal tubular epithelial cells and is one of the top 10 most common cancers worldwide 1. In USA, RCC incidence rates have increased by 2.421% per year and reached plateau since 2008 2. Although most detected lesions are small tumors, locally advanced disease continues to be diagnosed in a notable proportion of patients, with up to 17% of patients harboring distant metastases at the time of diagnosis 3. The 5-year relative survival rates through surgical treatment and adjunctive therapy have shown some improvement; however, the overall prognosis is still poor, particularly for metastatic RCC 4. Therefore, clarification of the detailed molecular mechanisms underlying RCC progression is critical for improving diagnosis and treatment of RCC.

Recent studies increasingly consider cancer a complex system that includes not only the cancer cells but also the tumor microenvironment (TME) that supports tumor growth. TME consists of different cell types including tumor cells, fibroblasts, endothelial cells, and immune cells, as well as non-cellular components 5. Macrophages are common immune cells in TME, enhancing tumor cell invasion and metastasis, angiogenesis, and extracellular matrix remodeling, while inhibiting the antitumoral immune surveillance. Increase in the number of infiltrating macrophages contributes to the tumor inflammatory microenvironment, resulting in the development of the cancer 6. Macrophages are typically categorized into two well-established polarized phenotypes, pro-inflammatory (M1) and anti-inflammatory (M2) macrophages 7. In the early stage of tumor development, the majority of macrophages are manifested as an M1 phenotype, which plays a role in inhibiting tumor growth. With the continuous progression of tumor cells, macrophages gradually transit to M2 phenotype, promoting tumorigenesis and progression 8. Researchers found that tumor-associated macrophages (TAM) display M2 macrophage characteristics and produce anti-inflammatory cytokines such as interleukin (IL)-10, IL-13 and transforming growth factor-β (TGF-β), promoting tumor initiation, growth, progression, metastasis, and immune evasion 9-12. Metastasis is the leading cause of cancer treatment failure and cancer-related mortality, cells to be metastasized under to EMT, and loss of the cell-cell contact 13. Tight junction protein is the first blocker in the inhibition of EMT or metastasis. Loss of tight junction leads to increase the permeability and hence secretion of exosome increased, which potentiate the communication of cancer cell and tumor microenvironment (TMA). TGF-β signaling or hypoxia plays an important role in the regulation of renal clear cell carcinoma. For example, loss of claudin-2 regulates the Hippo signaling and nuclear localization of YAP and induces EMT 14.

Exosomes (30-100 nm) are array of membranous vesicles under physiological conditions as well as in disease states released by eukaryotic cells 15. Exosomes are rich in protein, mRNA and miRNA, lncRNA et al. It transmits the significant biological messages to adjacent or distant cells, and then triggers the signal transduction inside the specific receptor cells. Therefore, exosomes are considered as important signal carriers to mediate the crosstalk between different cells. A recent study found that RCC-derived exosomes carry a new lncRNA, named lncRNA activated in RCC with sunitinib resistance (lncARSR), which enhances sunitinib resistance in RCC by the upregulation of AXL/c-MET and the activation of STAT3, AKT, and ERK signaling 16. Tumor cell-derived exosomes have been shown to reshape TME and differentiate macrophage between M1 and M2 polarization statuses 15, 17. Previous studies have found that exosomes derived from hypoxic epithelial ovarian cancer cells deliver microRNAs to macrophages and elicit transition into a tumor-promoted phenotype- M2 macrophage 18. Nevertheless, some other studies showed that certain tumor-derived exosomes can facilitate M1 macrophage transition and mediate pro-inflammatory responses 19.

The roles of exosomes and its cargo lncARSR in macrophage polarization and tumor development in RCC are still poorly understood. In this study, we revealed that RCC-derived exosome could carry the high levels of lncARSR, which induces the transformation of macrophage phenotype from M1 to M2 and promotes the cytokine secretion, phagocytosis and angiogenesis, thus significantly promoting the development of tumors. Furthermore, as a competitive endogenous RNA of miR-34/miR449-5p, lncARSR promotes the macrophage polarization through activation of the STAT3 pathway.

## Materials and methods

### Cell culture

The human RCC cell lines Caki-1, ACHN, 786-O cell line were obtained from Procell life Science & Technology Co., Ltd. The THP-1 was obtained from Shanghai Sixin Biological Technology Co., LTD. Caki cells were cultured in RPMI-1640 medium (HyClone, SH30809.01B) with 10% fetal bovine serum (FBS, Gibco, 10099141). Testing for mycoplasma contamination in cell cultures was routinely performed at least once every 3 months. Cell lines were maintained in culture for no more than 10 passages. The cell culture medium was super centrifuged at 100,000×g for 20 h to obtain exosome-free medium. The Caki cells were exposed to 20% O_2_ conditions. The THP-1 cells were cultured in RPMI-1640 medium (HyClone, SH30809.01B) with 10% fetal bovine serum (FBS, Gibco, 10099141). Macrophage generation and differentiation from THP-1 cells co-cultured with RCC cell lines after treated with 100 ng/ml PMA (Sigma-Aldrich, St. Louis, MO, USA) for 24 h.

### Flow cytometry assay

Macrophages were suspended in phosphate-buffered saline (PBS), followed by incubation with 100 μl Binding Buffer and FITC-labeled Annexin-V (20 μg/ml) 5 μl/sample for 30 min in the dark. Then 5μl of PI (50 μg/ml) was added in each sample for 5 min in the dark, followed by addition of 400 μl Binding Buffer. The samples were subjected to flow cytometric analysis within 1 h by FACScan.

### Exosome isolation

To isolate exosomes, human renal cancer cell line Caki-1 was cultured in exosome free RPMI-1640 for 24 h. We collected and centrifuged the supernatants two times (1000 g, 10 min and 3000 g, 30 min) to deplete the cells or fragments, followed by addition of Total Exosome Isolation Reagent (Life Technologies, 4478359) overnight and centrifugation for 10000 g for 1h at 4 °C. Exosomes were resuspended in PBS and stored at -80 °C. The concentration of exosomes was detected using a BCA Protein Assay. To detect RCC cells- derived exosomes when added in macrophages, exosomes were labeled with D384 (Invitrogen, S-32703), which is a phospholipid membrane dye (red). The macrophages were stained with DAPI (Sigma, D9542). After exosomes were incubated with macrophages at 37 °C for 2 h, live cell images were acquired with a Zeiss LSM 510 laser scanning confocal microscope.

### Electron microscopy

Two methods were used to identify exosomes, including electron microscopy and western blotting. As for electron microscopy, exosome pellets were re-suspended in PBS and dropped onto a carboncoated copper electron microscope grid. The exosomes were observed under a Tecnai G2 F20 ST Transmission Electron Microscope. At the same time, we detected exosome biomarkers by western blotting, including the tetraspanin molecule CD63 (Abcam, Rabbit pAb, ab216130) and CD81 (Abcam, Rabbit mAb, ab109201), and GAPDH (CST, Rabbit mAb, 5174) was used the internal control.

### Tissue samples

The ethical approval of this study was acquired from the Ethics Committee of TongDe Hospital of Zhejiang province, and written informed consents were obtained from all participants. A total of 8 pairs of RCC and adjacent normal renal tissue samples from patients who received surgical treatment were collected for this study between January 2019 and December 2020. Tissue samples were all snap-frozen in liquid nitrogen and preserved at -80 °C.

### Western blot

Cells were lysed in RIPA lysis buffer, then the collected cell protein was separated on 12% SDS-PAGE and transferred onto PVDF membranes. After the PVDF membrane was blocked by 5% skimmed milk, the diluted primary antibodies Abcam (Cambridge, MA) was added at 1:1000 and incubated all night. Following three washes in TBST, membranes were probed with the diluted secondary antibodies at 1:5000 (Abcam) for 2 h. ECL detection system was used for monitoring signals according to the manufacture's manual (Pierce, Rockford, IL).

### Tube-formation assay

Matrigel was evenly distributed to every well in a 24-well plate for 30 min at 37 °C. RCC cells at early passage were prepared after transfection in serum-free medium and 100 μl of 2*10^5^ cell suspension were added into each well. Tube formation was assessed under the microscope statistics by tube number.

### LncRNA Plasmid and siRNA transfection

Plasmid transfection: Cells were seeded in a 6-well plate at about 3×10^5^. Cells were transfected when reaching about 80% confluency. 2 μg plasmids (GenePharma, China) were transfected into cells with Liposome 2000 and cultured in Opti-MEM medium for 6-8 hours, and then cultured in normal medium.

SiRNA (GenePharma, ShangHai, China) Interference: The Caki-1 cells were seeded in a 6-well plate about 2×10^5^. The siRNA (final concentration 100 nM) was transfected into the cells with Lipofectamine 2000 (Invitrogen, California, USA). Lipofectamine was diluted well first (5 μl/ well). The RNA was diluted at the calculated concentration and incubated for 5 min respectively. The lipofectamine and RNA were mixed and incubated for 15 min in Opti-MEM (Gibco Company, Massachusetts, USA). The mixture was added into cells and cultured in an incubator for 6-8 hours before the culture medium was changed to fresh new culture medium.

### Reverse transcription quantitative real-time polymerase chain reaction (RT-qPCR)

The total RNA was isolated from cells by using the Trizol Kit in line with specification (Invitrogen) for reverse transcription using Takara RT reagent (Takara, Shiga, Japan). Quantitative analysis was implemented via StepOne Plus Real-Time PCR System (Applied Biosystems, Foster City, CA). All results were processed with 2^-ΔΔCT^ method after normalizing to ACTIN or U6.

### Enzyme-linked immunosorbent assay (ELISA)

Macrophage generation and differentiation from THP-1 cells co-cultured with RCC cell lines. RCC cells were seeded in the upper insert of a six-well Transwell apparatus (0.4 μM pore size, Corning, Lowell, MA) while THP-1 with 320 nM PMA in the lower chamber. After 72 h, cell supernatants were collected and processed using affymetrix eBioscience ready-set-go ELISA kits according to manufacturers' instructions to detect THP-1 cell expression of IL-10 (cat# 88-7105), TGF-β (cat# 88-8350), TNF-α (cat# 88-7324) and IL-1β (cat# 88-7013-86). The 2-tailed Student's t test was used to determine statistically significant, *p* values less than 0.05 is statistically significant for the differences in cytokine expression between groups.

### Immunohistochemical staining

Tumor tissues from mice were obtained and fixed in formalin. The tissues were embedded into paraffin blocks and then cut into 4 μm-thick sections. After mounting on slides, immunohistochemistry (IHC) was performed. The primary antibodies mouse anti- F4/80 (1:200, Cell Signaling Technology), Ki67 (1:200, Abcam) and mouse anti-CD206, Human-CD68, CD206 (1:100, Santa Cruz Biotechnology) expression were shown by horseradish peroxidase-diaminobenzidine (HRP-DAB) immunostaining.

### Bioinformatics analysis

TCGA KIRC expression profiles including macrophages expression profiles, were downloaded from the TCIA database (https://www.tcia.at/home). ENCORI (https://starbase.sysu.edu.cn/index.php) and miRwalk (http://129.206.7.150/) databases were used to search for Mir-34a-5p, miR-34b-5p, miR-34c-5p, miR-499a-5p, miR-34a-3p, miR-34b-3p, miR-34c-3p, miR-499a-3p, miR-499b-3p may regulation genes, and will be treated as income target genes do intersection, using Metascape (http://metascape.org/gp/index.html) according to intersection analysis may regulate related signaling pathway. The differential expression and correlation analyses were performed using GraphPad Prism 7 software (GraphPad Software, USA).

### Animal models of tumors

Specific pathogen-free (SPF) female BALB/c nu/nu mice (6-8 weeks old) were purchased from Nanjing GemPharmatech. All of the protocols were approved by the Committee for Ethical Affairs of Tongde Hospital of Zhejiang Province (Zhejiang, China), and the methods were carried out in accordance with the approved guidelines. In the Caki group, Caki cells were first infected with luciferase-GFP lentivirus and then injected into renal capsule of nude mice (1×10^6^/20 μl). In the Exo + Caki group, enriched tumor exosomes were injected into caudal vein of nude mice for pretreatment. After three weeks of intervention, Caki-1 cells were implanted under renal capsule. In the NC group, normal control mice were used. The image analyses were performed using PerkinElmer IVIS. All of the mice were bred in the Animal Research Center of Zhejiang Academy of Traditional Chinese Medicine (Zhejiang, China) in compliance with the Guide for the Care and Use of Laboratory Animals.

### Statistical Analysis

The experimental data were presented as the mean ± SD. Statistical analysis was performed using Prism 8.0. Two-tailed Student's t-tests and one-way ANOVA were used to analyze the two groups difference or multiple group comparisons, respectively. The value of P< 0.05 was considered to be statistically significant.

## Results

### High tumor-associated macrophages (TAM) infiltration is associated with poor prognosis of renal carcinoma

**Figure 1:**
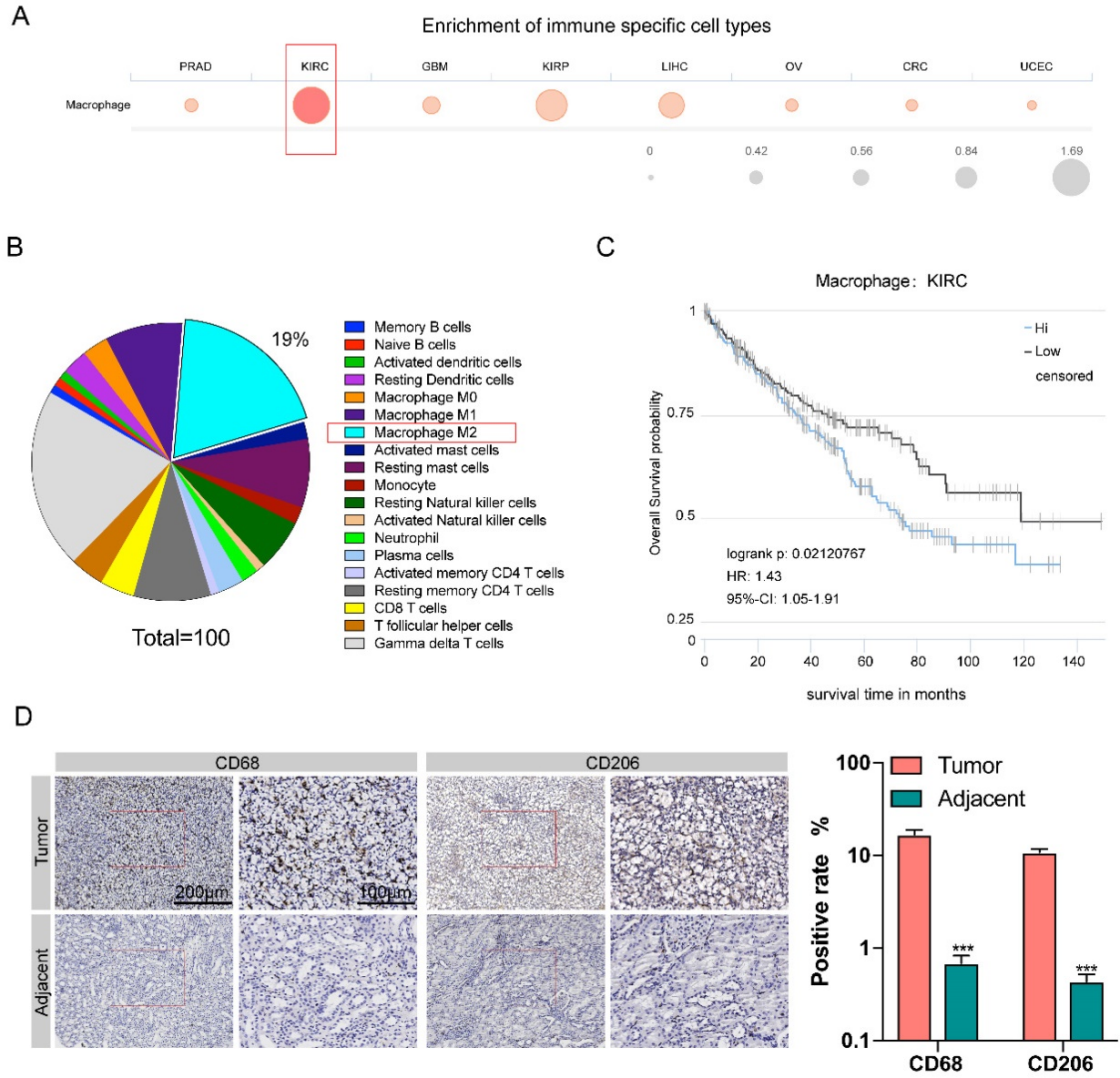
** High macrophage infiltration in KIRC correlates to worse prognosis. A.** Comparison of the degree of immune cells infiltration among eight different kinds of cancers. **B.** Proportion of different types of immune cells resided in KIRC tissue. **C.** The survival curve of KIRC patients with low and high degree of macrophage infiltration in the KIRC tissue. **D.** The expression level of CD68 and CD206 in cancer tissues was higher than para-cancerous tissues (p<0.001).

The Cancer Immunome Atias Database (TCIA: https://tcia.at/home) provides results of comprehensive immunogenomic analyses of next generation sequencing data (NGS) data for 20 solid cancers from the Cancer Genome Atlas (TCGA) and other data sources. We analyzed data from TCIA database and compared the degree of immune cells infiltration in eight different kinds of cancers, including prostate adenocarcinoma (PRAD), kidney renal clear cell carcinoma (KIRC), glioblastoma multiforme (GBM), kidney renal papillary cell carcinoma (KIRP), liver hepatocellular carcinoma (LIHC), ovarian serous cystadenocarcinoma (OV), colorectal cancer (CRC), and uterine corpus endometrial carcinoma (UCEC). Among these eight types of tumors, the number of macrophages infiltration of kidney renal clear cell carcinoma (KIRC) tissue was the largest** (Figure 1A)**. In KIRC tissues, M2 macrophages are the second most abundant type of immune cells (after Gamma delta T cells), accounting for 19% of all immune-related cells **(Figure 1B)**. A Kaplan-Meier survival analysis was used to investigate the correlation between macrophage infiltration and patient survival through the TCIA database, it was shown that the degree of macrophage infiltration is closely related to RCC prognosis. The 10-year survival rate of macrophage with high infiltration was significantly lower than that with low infiltration (P=0.021) **(Figure 1C)**.

Next, we compared the amount of M2 macrophages between renal carcinoma tissues and para-cancerous tissues collected clinically. We examined the CD68 expression for pan-macrophage and CD206 expression for M2 macrophage by immunohistochemistry. RT-qPCR showed the expression of CHI3L1, IL-10, RETNLB (Fizz1), and Arg1, to confirm the M2 macrophages** (Supplementary Figure 1A)** The results showed that the expression level of both CD68 and CD206 were significantly increased in cancer tissues compared with paracancerous tissues **(Figure 1D)**.

### Exosomes derived from renal cancer cells promote macrophage polarization and tumor development *in vivo*

**Figure 2:**
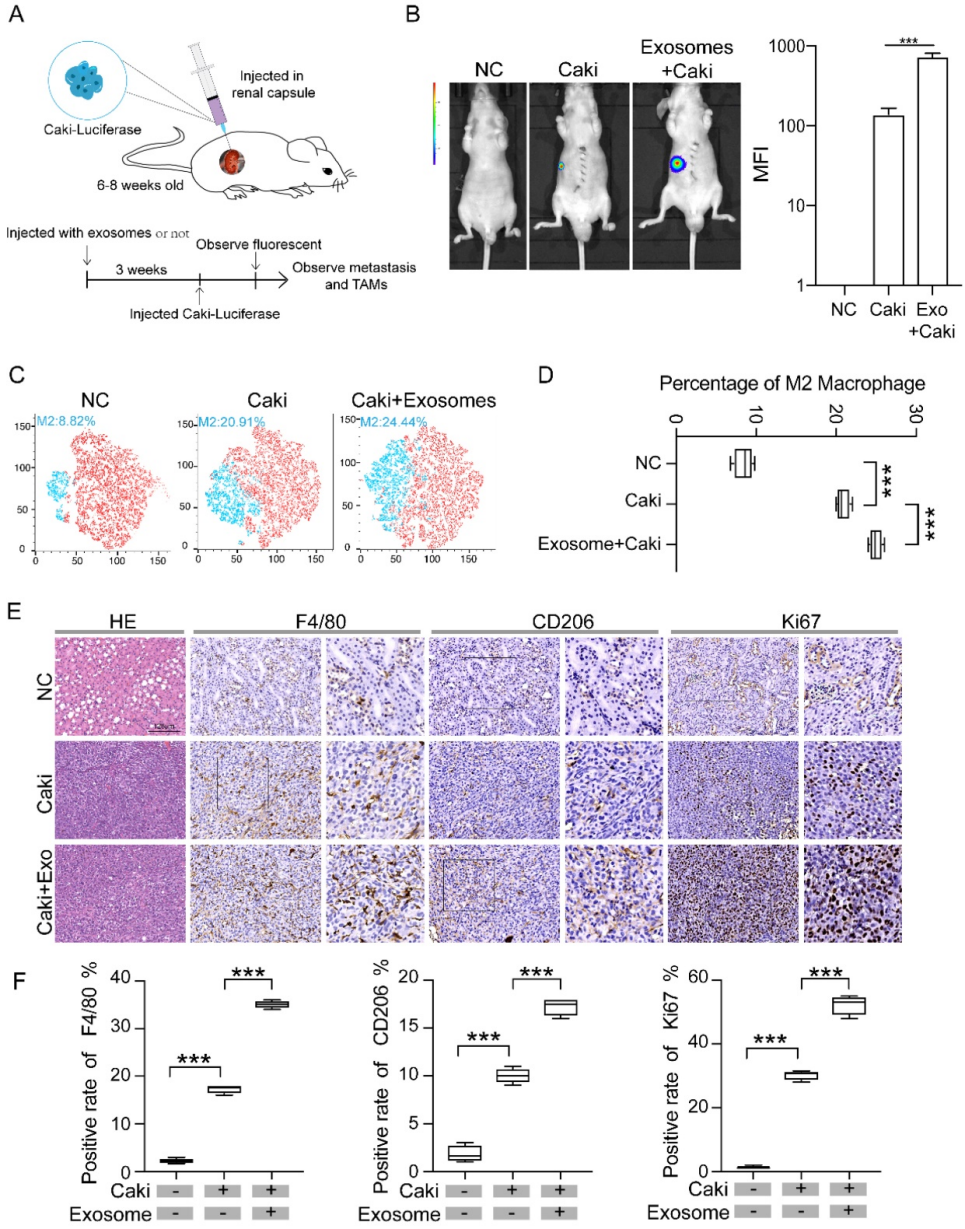
** Exosomes derived from renal cancer cells promote macrophage polarization and tumor development *in vivo*. A.** Schematic of RCC nude mice model. **B.** Comparison of the tumor volume among three groups: normal control, Caki only, and Exo+Caki. ***P<0.001. **C-D.** Flow cytometry analysis of macrophage phenotypes in mice groups treated or not treated with exosomes. **E.** Representative IHC images of tumor tissues from Caki and Exo+Caki mice or kidney from NC mice to demonstrate the expression of F4/80, CD206and ki67. ***P<0.001. **F.** Quantitative analysis of ISH of F4/80, CD 206 and ki67 among three groups. ***P<0.001.

We then constructed RCC mice models by injecting Caki-1 cells infected with luciferase-GFP letivirus into renal capsule of nude mice (1 × 10^6^/20 μl) **(Figure 2A)**. Three weeks after injection of Caki-1 cells, the RCC tumor can be visualized by fluorescence, while no fluorescence was detected in the normal control group without injection of Caki-1 cells **(Figure 2B)**. To test whether tumoral exosomes affect the tumor growth, enriched tumor exosomes derived from Caki-1 cells were injected into caudal vein of nude mice three weeks before the injection of Caki-1 cells. The tumor volume of mice group pretreated with tumor exosomes (Exo + Caki) was significantly larger than that of mice injected with Caki-1 cells only, indicating that RCC derived exosomes promote tumor development **(Figure 2B)**. We then examined if the macrophage phenotype is different in mice treated with tumor exosomes by flow cytometry analysis. The results revealed that the proportion of type M2 macrophage in the Exo+Caki group was significantly higher than that in the Caki group (24.44% vs 20.91%, p<0.001) **(Figure 2C-D)**. The expressions of F4/80 and CD206 were measured in the resected tumor tissues from tumor bearing mice or kidney from Negative Control group mice by immunohistochemical staining. The results indicated that both expression of F4/80 and CD206 in the tumor tissues were significantly higher in the Exo+Caki group than Caki group (p<0.001), suggesting that exosomes enhance the macrophage filtration into tumor. Additionally, the Exo+Caki group had remarkably increased expression of Ki67 protein that is associated with cell proliferation** (Figure 2E-F)**, suggesting that exosomes stimulate tumor cell proliferation.

### RCC-derived exosomes regulate macrophage polarization *in vitro*

Growing evidence has suggested that exosomes derived from different tumors can potentially alter the macrophage fate of differentiation 15 and promote cancer development, but the studies in RCC were still lacking. Tumor metastasis is a series of complex processes including cell invasion, angiogenesis, infiltration of blood vessels and invasion of neighboring and distant host organs. In addition to the changes of tumor cells themselves, more and more studies have focused on the communication and material delivery between cells in the tumor microenvironment. Recent evidence suggests that tumor-derived exosomes play an important role in mediating the intercellular communication between immune cells and tumor cells in the tumor microenvironment. These exosomes are involved in different stages of tumor development by promoting macrophage M2 polarization and thus enhancing tumor progression. To understand the role of RCC derived exosomes in TAM phenotypic conversion, we performed *in vitro* experiments by culturing macrophages (THP-1) with RCC cell (789-O, ACHN and Caki-1)-derived exosomes.

Firstly, we isolated the exosomes secreted by three different RCC cell lines, including 789-O, ACHN and Caki-1. The nanoscale exosomes (30-100 nM) were verified by electron microscope** (Supplementary Figure 1B).** To further confirm the identity of exosomes, Western blot was applied to measure the expressions of the exosomal marker CD63 and CD81. The results confirmed that exosomes were successfully isolated as CD63 and CD81 were enriched in the exosomes derived from all three RCC cell lines **(Supplementary Figure 1C)**.

**Figure 3:**
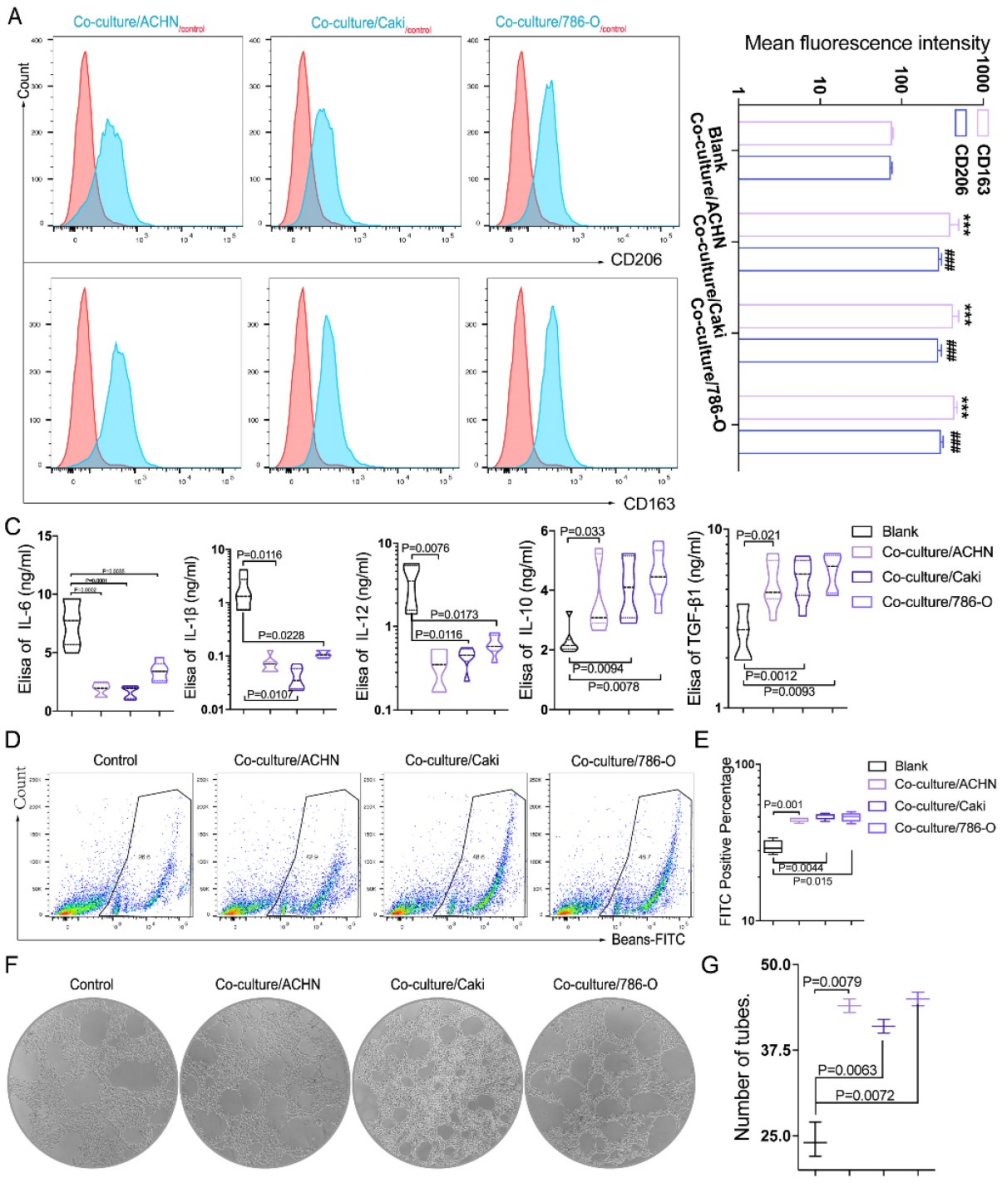
** RCC-derived exosomes regulate macrophage polarization *in vitro*. A-B.** Through flow cytometry, we found that compared with macrophages as the control group, the fluorescence intensity of CD163 and CD206 were significantly increased after RCC cell lines (789-O, ACHN and Caki-1) derived exosomes and macrophages co-cultivation (P<0.001) CD163:***P<0.001; CD206: ###P<0.001. **C.** The supernatants before and after the co-culture were collected, and the Elisa test showed that the expression of cytokines TGFbeta-1 and IL-10 increased, and IL-6, IL-12, IL-1beta decreased significantly after co-culture (p<0.05). **D-E.** The phagocytic ability of macrophages was significantly enhanced after co-culture with RCC-derived exosomes (p<0.05). **F-G.** Through tube formation assay we found that the pro-angiogenic ability of macrophages was significantly enhanced after co-cultured with RCC-derived exosomes (p<0.01).

Then we cultured macrophages (THP-1) with exosomes. Through flow cytometry, we found that the fluorescence intensities of CD163 and CD206 were significantly increased in macrophages after treating with exosomes derived from three RCC cell lines (789-O, ACHN and Caki-1)** (Figure 3A-B),** suggesting that the exosomes can promote macrophage M2 polarization *in vivo*. The supernatants before and after the co-culture were collected, and the ELISA test showed that the expression of cytokines TGFbeta-1 and IL-10 increased, and IL-6, IL-12, IL-1beta decreased significantly after co-culture **(Figure 3C)**, indicating that more anti-inflammatory cytokines are secreted. The phagocytic ability of THP-1 was measured by flow cytometry after the CAKI-1 cells were labeled with FITC-beads for 30min. It was found that the phagocytic ability of macrophages was significantly enhanced after co-culture with RCC-derived exosomes** (Figure 3D-E)**. In addition, through tube formation assay we found that the pro-angiogenic ability of macrophages was significantly enhanced after co-cultured with RCC-derived exosomes **(Figure 3F-G)**. The results indicated that RCC derived exosome could promote the transformation of macrophages to M2 type, increase the secretion of cytokines, enhance the phagocytosis ability of macrophages, and induce angiogenesis.

### LncARSR in RCC derived exosomes was significantly high expression

**Figure 4:**
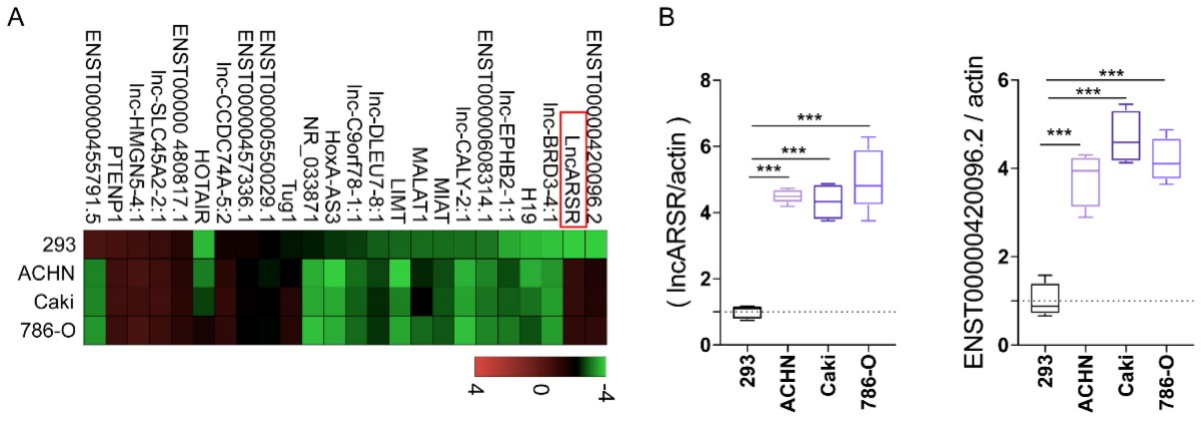
** LncARSR in RCC derived exosomes was significantly high expression. A-B.** lncARSR and ENST00000420096.2 were highly expressed in RCC derived exosomes by RT-qPCR verification (***P<0.001).

The main mechanism that tumor-derived exosomes modify macrophages polarization is that exosomes deliver a large number of biological cargos such as miRNAs and lncRNAs, etc., to the recipient macrophage cells. The lncRNAs were determined and measured by RT-qPCR. We found that lncARSR and ENST00000420096.2 were especially highly expressed in RCC derived exosomes **(Figure 4A-B)**. The increased expression of lncRNAs in the exosomes derived from the three different RCC cells were then verified by RT-qPCR.

### Exosomal lncARSR could induce phenotypic and functional changes of macrophages *in vitro*

**Figure 5:**
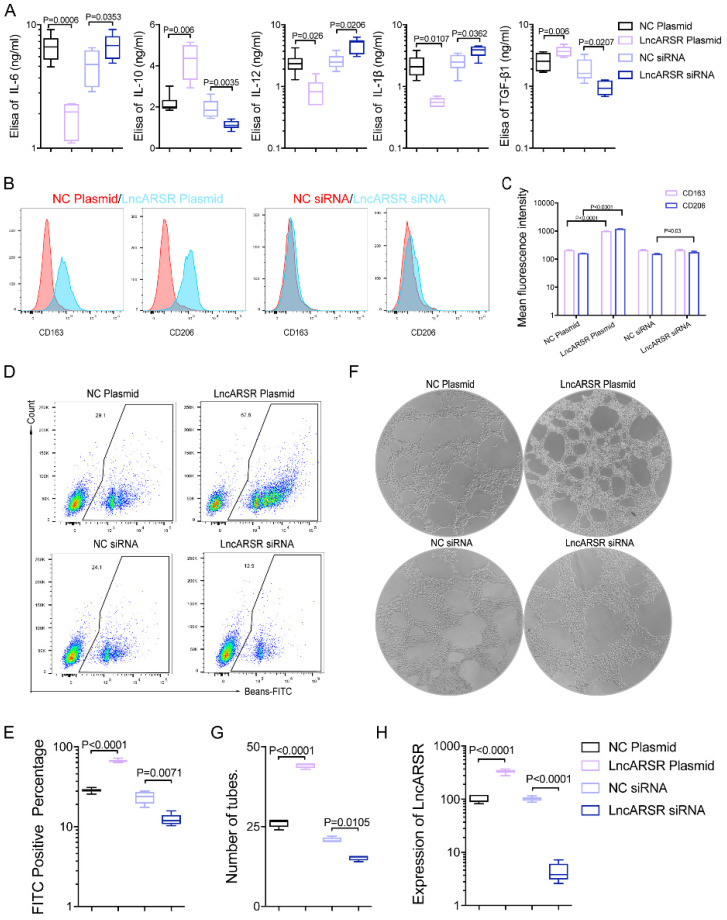
** Exosomal lncARSR could induce phenotypic and functional changes of macrophages *in vitro*. A.** LncARSR plasmid/NC siRNA (control group)/lncARSR siRNA was transfected into macrophages, and we found that compared with the control group, the ability of cytokines secretion by macrophages with lncARSR plasmid transfection was enhanced (p<0.01), and on the contrary the ability of cytokines secretion by macrophages with lncARSR siRNA transfection was weakened through Elisa testing(p<0.01). **B-C.** The fluorescence intensity of CD163 and CD206 expressed by macrophages transfected with lncARSR plasmid was significantly enhanced (p<0.01), and the fluorescence intensity of CD163 and CD206 expressed by macrophages transfected with lncARSR siRNA was significantly decreased by flow cytometry (p<0.05). **D-F.** The phagocytic ability of macrophages transfected with lncARSR plasmid was enhanced (p<0.001), and inversely the phagocytic ability of macrophages transfected with lncARSR siRNA were suppressed (p<0.01). **E-H.** The proangiogenesis ability of macrophages was enhanced after lncARSR plasmid transfection (p<0.001) and inversely was inhibited by lncARSR siRNA through tube formation assay (p<0.05).

To evaluate whether the increased exosomal lncARSR is involved in the macrophage polarization, we alter lncARSR levels in macrophages by overexpressing lncARSR or reducing its expression through siRNA. The results showed significantly increased levels of IL-10 and TGFβ1 secretion in macrophages overexpressing lncARSR, and reduced IL-10 and TGFβ1 secretion in macrophage treated with lncARSR siRNA. On the contrary the levels of IL-6, IL-12 and IL-β was reduced in macrophages overexpressing lncARSR, and increased in macrophage with decreased lncARSR** (Figure 5A)**. In addition, the macrophage polarization was assessed by measuring the fluorescence intensity of M2 macrophage marker CD163 and CD206 with flow cytometry. Overexpression of lncARSR in macrophages significantly enhanced CD163 and CD206 levels and decrease in lncARSR reduced the fluorescence intensity of CD163 and CD206 **(Figure 5B-C)**, suggesting that lncARSR stimulates the macrophage polarization. The phagocytic ability of macrophages transfected with lncARSR plasmid was enhanced, and inversely the phagocytic ability of macrophages transfected with lncARSR siRNA were suppressed **(Figure 5D-F)**. Lastly, the proangiogenic ability of macrophages was enhanced after lncARSR plasmid transfection and inversely was inhibited by lncARSR siRNA through tube formation assay **(Figure 5E-G)**. The results showed that exosome-transmitted lncARSR would contribute to the secretion of cytokines, the phagocytosis and proangiogenic ability of macrophages.

### LncARSR activates the STAT3 pathway to facilitate macrophage M2 polarization

We next explored the potential downstream gene or signaling pathways that can be regulated by lncARSR. LncARSR is a relatively new-found exosomal lncRNA and only pathways are associated with lncARSR in the gene database at present. Previous studies have reported that lncARSR acts as a molecular sponge for miR-34 and miR-449 to regulate the expression of related downstream genes 16. Through bioinformatics analysis, we identified the potential target genes downstream of miR-34 and miR-449. We then carried out pathway enrichment analysis and GO enrichment analysis, and discovered the signaling pathways related to macrophage polarization **(Supplementary Figure 2A-D)**. Through the bioinformatic analysis, we hypothesized that lncARSR might induce macrophages phenotype shift to TAM through regulation of the STAT3 signaling pathway.

**Figure 6:**
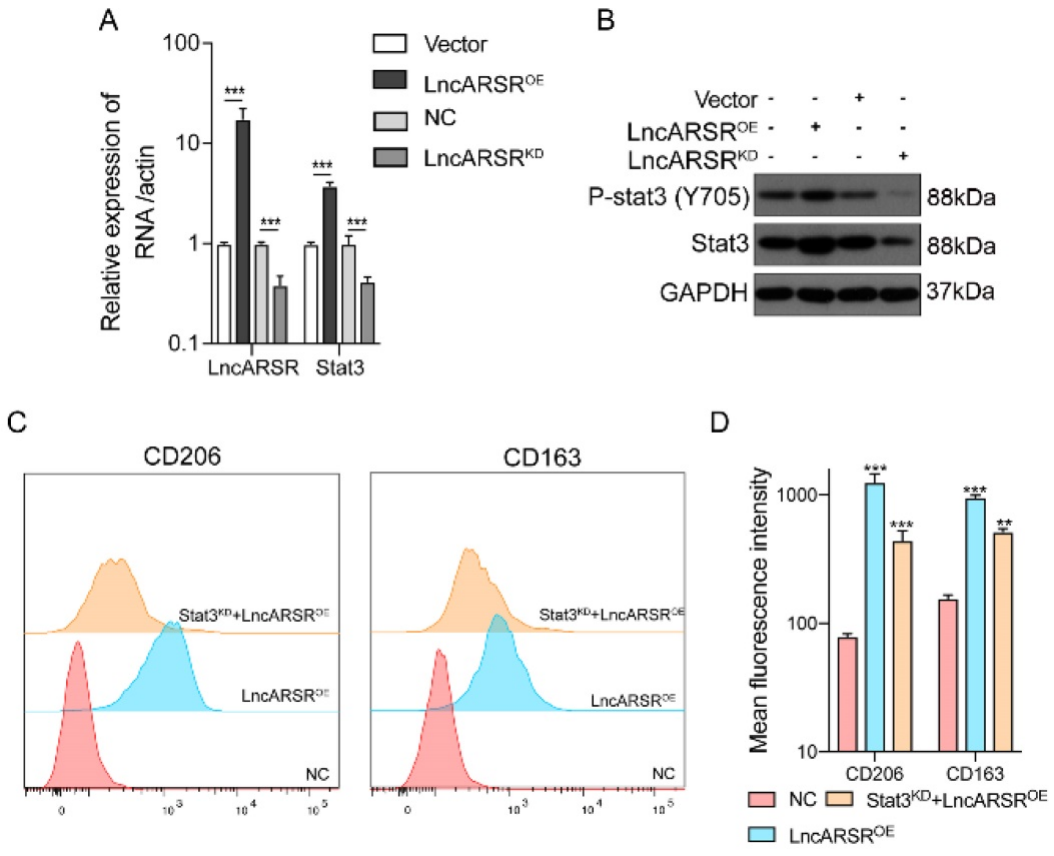
** LncARSR activates the STAT3 pathway to facilitate macrophage M2 polarization. A-B.** After overexpression or knockdown of lncARSR, the expression level of lncARSR and STAT3 were detected by RT-qPCR and Western blot. The expression level of STAT3 was higher in lncARSR overexpression group than the knockdown one with synchronous tendency (p<0.001). **C-D.** After the target gene STAT3 was knockdown by siRNA, CD206 and CD163 MFI of STAT3 knockdown group was lower than lncARSR overexpression group (p<0.01). STAT3 knockdown could rescue the effect of lncARSR. **P<0.01, ***P<0.001 vs NC.

We then investigated whether the STAT3 expression can be regulated by lncARSR. After overexpression or knockdown of lncARSR, the expression level of lncARSR and STAT3 were measured by RT-qPCR and Western blot. The mRNA and protein level of STAT3 in macrophage was significantly higher in lncARSR overexpression group, and reduced in cells with lncARSR knockdown **(Figure 6A-B)**. More importantly, the lncARSR-mediated increases in CD206 and CD163 levels were significantly suppressed by STAT3 silencing, further supporting our hypothesis that lncARSR-induced phenotypic changes of TAM are mediated through the STAT3 pathway** (Figure 6C-D)**.

### LncARSR promotes tumor growth and induces macrophage polarization *in vivo*

**Figure 7:**
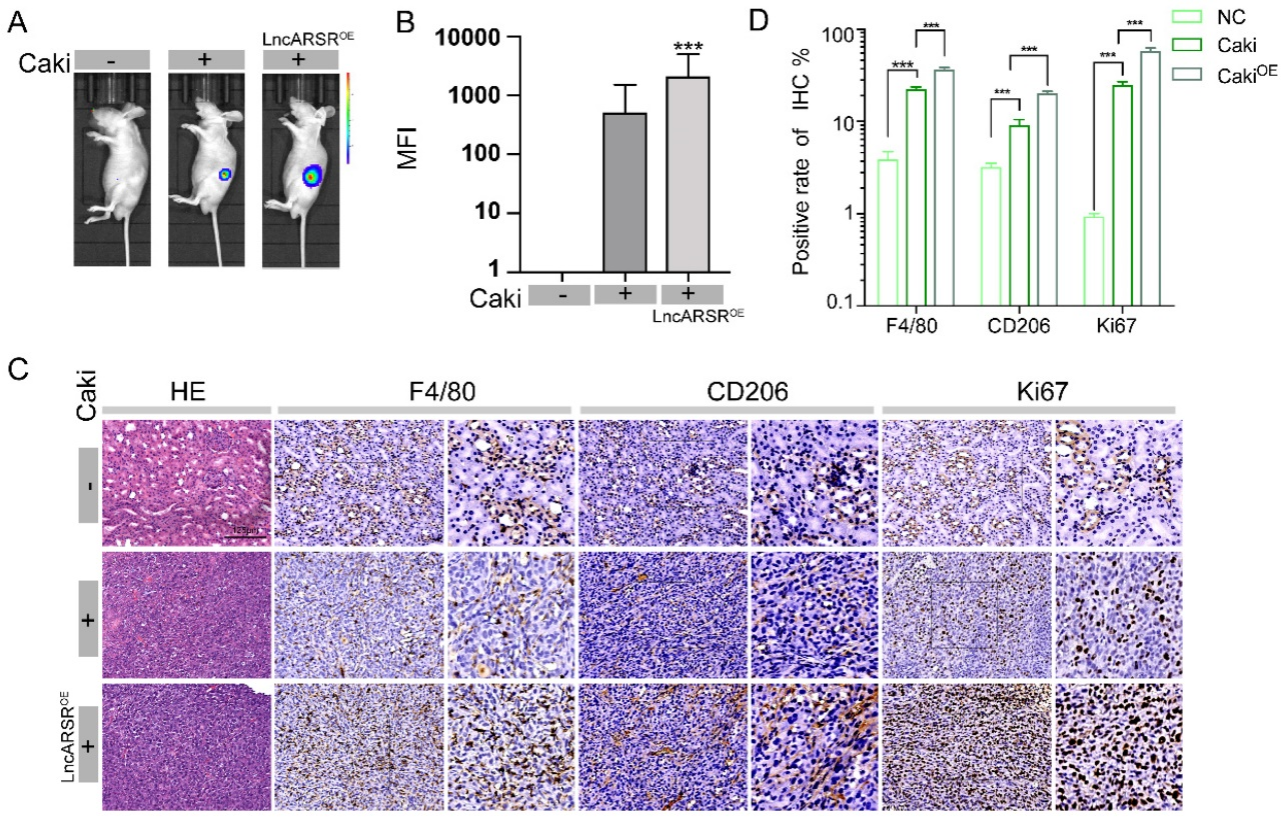
** LncARSR promotes tumor growth and induces macrophage polarization *in vivo*. A-B.** Through animal fluorescence quantitative analysis, we found that the MFI (mean fluorescence intensity) value of nude mouse tumors in lncARSR-overexpression group was significantly higher than that of Caki implant group and control group (***P<0.001). **C-D.** We detected the expression of F4/80, CD206 and Ki67 in tumor specimens of nude mice by immunohistochemistry. We found that the tumor volume of nude mice was significantly increased and the expression levels of F4/80, CD206 and Ki67 were significantly increased by immunohistochemistry in the lncARSR overexpression group (***P<0.001).

After validating that lncARSR can promote macrophage polarization *in vitro*, we next examined its roles *in vivo* using the RCC orthotopic tumor model. Similar to the procedure in Figure 3A, Caki-1 cells were injected into renal capsule of nude mice at a dose of 1*10^6^ and tumor formation in nude mice was observed three weeks later by GFP fluorescence imaging. The mice injected with Caki-1 cells infected with lncARSR lentivirus had significantly higher mean fluorescence intensity (MFI) than the mice injected with Caki-1 cells only and normal control group **(Figure 7A-B)**. We measured the expression of F4/80, CD206 and Ki67 in tumor specimens of nude mice by immunohistochemistry. We found that the tumor volume of nude mice was significantly increased and the expression levels of F4/80, CD206 and Ki67 were significantly increased by immunohistochemistry in the lncARSR overexpression group **(Figure 7C-D)**. These results showed that lncARSR could significantly promote tumor development and induce macrophage polarization *in vivo*.

### LncARSR promotes the development of RCC through transmit by exosomes to macrophages

We have clearly demonstrated that lncARSR can promote macrophage polarization and RCC tumor growth. We sought to examine whether lncARSR is the major factor mediating exosome-induced RCC development *in vivo*. The Caki-1 cells were treated with siRNA to reduce the levels of lncARSR. Then the total exosomes were isolated from supernatants of treated with or without lncARSR siRNA. Exosomes (4-5 μg/mouse, once every other day) were continuously injected intravenously into nude mice at exosome pretreatment group for 3 weeks. The orthotopic transplantation model of nude mice was then established as showed in Figure 3A by injection of Caki-1 cells into renal capsule, and the nude mice were divided into four groups: no pretreatment group, exosome pretreatment group, lncARSR-knockdown exosome pretreatment group, and the normal control.

**Figure 8:**
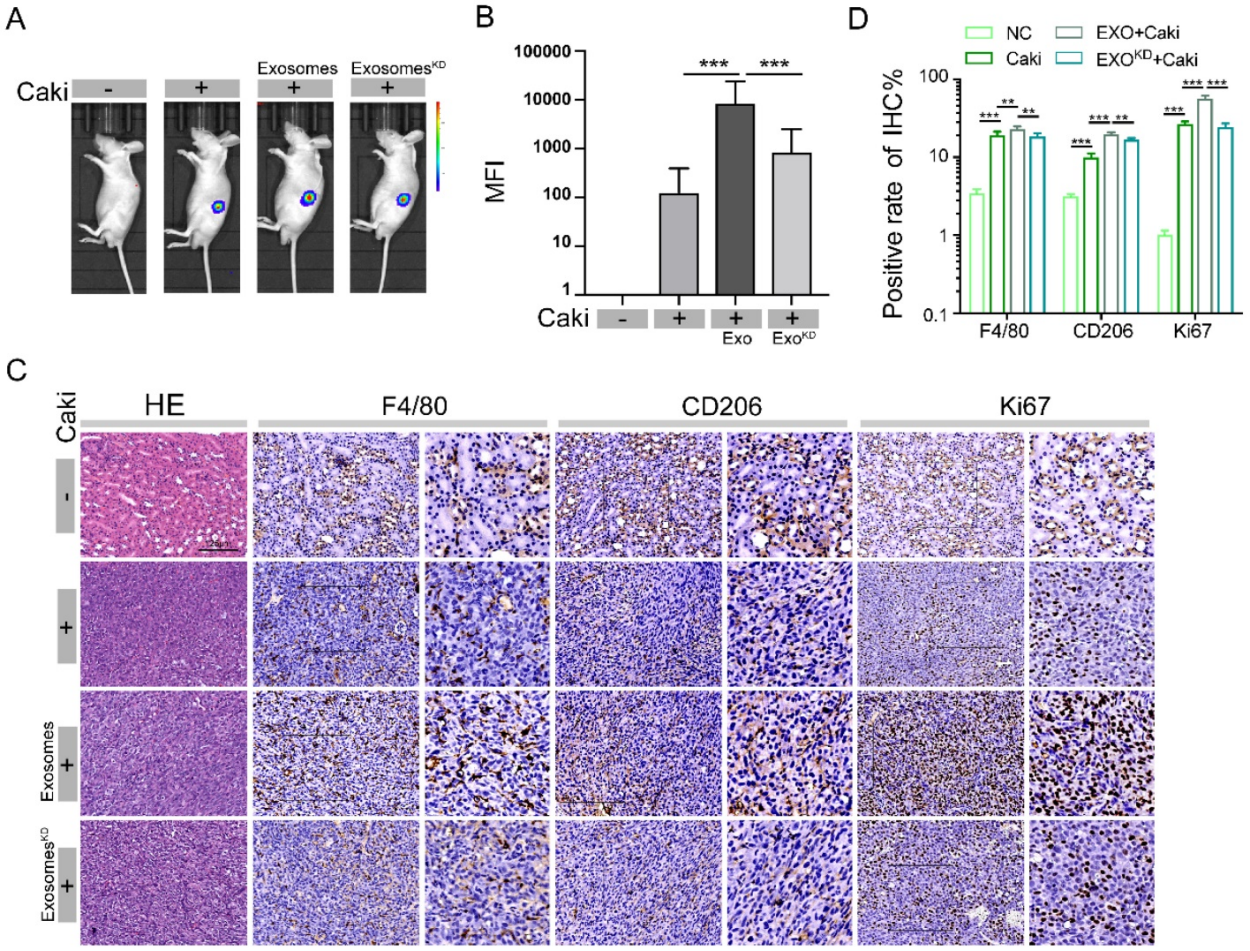
** LncARSR promotes the development of RCC through transmit by exosomes to macrophages. A- B.** Through animal fluorescence quantitative analysis, we found that the MFI of nude mouse tumors in exosome pretreatment group was significantly higher than the MFI value in no pretreatment group, lncARSR-knockdown pretreatment group, and the control after 3 weeks pretreatment (***P<0.001). **C-D.** We detected the expression of F4/80, CD206 and Ki67 in tumor specimens of nude mice by immunohistochemistry. The expression levels of F4/80, CD206 and Ki67 of tumor tissue were higher in exosome pretreatment group than those in lncARSR-knockdown pretreatment group, no pretreatment group and the control (**P<0.01, ***P<0.001).

Through animal fluorescence quantitative analysis, we found that the MFI of nude mouse tumors in exosome pretreatment group was significantly higher than the MFI value in no pretreatment group, while lncARSR-knockdown exosome pretreatment group had significantly reduced MFI value **(Figure 8A-B)**, indicating, the tumor size was significantly larger in exosome pretreatment group than lncARSR-knockdown pretreatment group and no pretreatment group, suggesting that the protumor effects of exosomes are mediated through lncARSR. We then measured the expression of F4/80, CD206 and Ki67 in tumor specimens of nude mice by immunohistochemistry. The expression levels of F4/80, CD206 and Ki67 of tumor tissue were higher in exosome pretreatment group than control group, while lncARSR-knockdown pretreatment reduced expression of F4/80, CD 206 and Ki67 **(Figure 8C-D)**, which indicates that lncARSR is also responsible for the macrophage polarization and increased tumor proliferation mediated by exosomes. Our results reveal that RCC derived exosomes promote tumor growth and macrophage polarization by carrying lncARSR cargo.

## Discussion

As one of the top 10 most common cancer, RCC incidence rates remain high, especially in developed country. Studies have revealed an enrichment of macrophages in RCC 20 **(Figure 1)**, therefore, it is important to understand the crosstalk between RCC tumor and macrophages. We discovered that RCC-derived exosomes carry a high amount of lncARSR, which acts on local macrophages and activates the STAT3 pathway, resulting in the transformation of macrophage phenotype from M0/1 to M2 **(Supplementary Figure 3)**. Such macrophage polarization results in secretion of a large number of anti-inflammatory factors (such as IL-10 and TGF-beta1, etc.), creating local microenvironment more suitable for tumor metastasis and the formation of pre-metastatic lesions.

Studies over the past two decades have revealed that the TME is an important determinant of tumor behavior. The components of the TME include local stromal cells, such as resident fibroblasts and macrophages, and distantly recruited cells such as endothelial cells, myeloid and lymphoid cells, bone marrow-derived precursor cells, and circulating platelets 21. Macrophages show a surprising degree of plasticity in functional reprogramming and adopt either pro- or anti-inflammatory phenotypes in response to environmental stimuli within TME 22. When exposed to certain cytokines such as IL12, TNF, interferon gamma (IFNG), and microbe-associated molecular patterns (MAMPs) such as bacterial lipopolysaccharide (LPS), or other Toll-like receptor (TLR) agonists, macrophages acquire a pro-inflammatory (M1) state. Conversely, IL4, IL5, IL10, IL13, CSF1, transforming growth factor beta 1(TGF β1), and PGE2 all promote macrophage polarization toward an anti-inflammatory (M2) state and suppress the activity of effector T-cells and other immune cells 23.

Increasing evidence demonstrates that macrophage transitions from M1 to M2 phenotypes might boost cancer initiation and progression by promoting cell proliferation, metastasis, drug reliance, and immune evasion 24, 25. Previous studies also showed that CD68 and CD206 are markers of macrophages and M2 macrophages and increased density of TAMs is associated with poor survival of patients 26. Consistent with previous studies, we found that the infiltration of macrophages was high in KIRC tissues, and the degree of infiltration was correlated to the poor prognosis of tumors by analyzing the TCGA database. We further confirmed the presence of high number of macrophages in cancer tissues collected from renal carcinoma patients by measurement of CD68 (macrophage marker) and CD206 (M2 macrophage marker).

Exosome is a nanoscale extracellular vesicle with spherical shape surrounded by lipid bilayers 27. Exosome plays important roles as the vesicular cargo for carrying and transferring molecular mediators for cell-cell communications and signal transduction 28. Recent studies investigating tumor secreted exosomes found that exosome can regulate tumor-associated fibroblast (CAF) and tumor-associated macrophage (TAM) in TME by carrying specific small molecule substances, thus affecting the occurrence, development and metastasis of tumors. For example, exosomes secreted by bladder cancer cells activate and drive differentiation of healthy fibroblasts to CAFs through the TGFβ-mediated SMAD pathway 29, and melanoma-derived exosomal miR-125b-5p targets lysosomal acid lipase A (LIPA) in macrophages and promotes phenotypic changes in macrophages 10. Some scholars also have found that under hypoxic conditions, EOC cell-derived exosomes deliver MiR21-3P, miR-125b-5p and miR-181d-5p to induce M2 macrophage polarization, which promotes EOC cell proliferation and migration macrophages and elicit transition into a tumor-promoted phenotype- M2 macrophage. Through both *in vivo* and *in vitro* experiments, we found that RCC-derived exosome can increase the proportion of M2-type macrophages, which suggests that exosome can promote the phenotypic transformation of macrophages and enhance the ability of promoting cancer in renal cancer cells.

Exosome-Transmitted lncARSR was first identified as a competitive endogenous RNA of miR34/miR449 promoting sunitinib resistance and high levels of lncARSR in RCC patients correlated with poor response to sunitinib therapy in renal cell cancer 16. Another study showed that high lncARSR expression correlated with poor prognosis of RCC patients 30. However, it is still unclear how lncARSR is related to the RCC development. In this study, *in vivo* and *in vitro* experiments showed that exosome-transmitted lncARSR could promote the transformation of macrophages to M2 type, the secretion of IL-10, TGF-β1 while inhibit the secretion of IL-6, IL-1β and the differentiated secretion of anti-inflammatory and pro-inflammatory cytokines was also consistent with the characteristic behavior of type M2 macrophages 23. Exosome-transmitted lncARSR could also enhance the phagocytosis ability of macrophages, induce angiogenesis, and thus promote the formation of local tumor microenvironment. In addition, we identified that signal transducer and activator of transcription 3 (STAT3) is a new downstream signaling pathway regulated by lncARSR.

STAT3 is a transcription factor that is activated downstream of a broad range of receptors particularly interleukin-6 (IL-6) family. STAT3 is the key regulator of cell proliferation, survival and apoptosis and is constitutively activated in most human cancers. It is reported that macrophage-secreted NGAL by the regulation of STAT3 shapes the pro-tumorigenic macrophage phenotype to contribute to breast cancer progression 31, 32. In renal cell carcinoma, earlier studies have shown that increased STAT3 activation has been associated with progression of pathological stages and worse prognosis 33-35. Our result revealed that STAT3 silencing significantly reduced lncARSR-mediated macrophage polarization and this finding was consistent with previous literature reports. It is not known if this lncARSR-miR34/miR449-STAT3 signaling pathway is involved in other types of cancers. Future work will be conducted to investigate whether reduction in lncARSR or STAT3 levels might inhibit other cancer progression by boosting the immune response.

In conclusion, our study discovers that lncARSR acts as the signaling molecule delivered by RCC-derived exosomes to induce macrophages polarization by activating the STAT3 signaling pathway, and changes its cytokine secretion and phagocytosis ability, thus promoting the occurrence and development of tumors. The lncARSR-miR34/miR449-STAT3 signaling pathway is a new therapeutic target for the prevention and treatment of RCC progression.

## Supplementary Material

Supplementary figures.
