# Supplementary material for: Renal cell carcinoma-derived exosomes deliver lncARSR to induce macrophage polarization and promote tumor progression via STAT3 pathway
Source: Int J Biol Sci. 2022 May 1;18(8):3209–22. doi: 10.7150/ijbs.70289 (PMC9134902; doi:10.7150/ijbs.70289)
Supplement: Supplementary file 1 — Supplementary figures. [file ijbsv18p3209s1.pdf]

Supplementary Figure 1

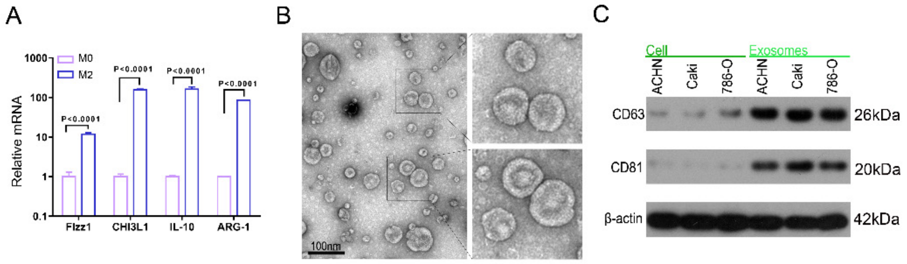

A. RT-qPCR determined the expression of CHI3L1, IL-10, RETNLB (Fizz1), and Arg1. B. The electron microscopic image of RCC-derived exosomes. C. The expression of CD63 and CD81 in three RCC cell lines (ACHN, Caki-1, 786-O) through Western blotting.

Supplementary Figure 2

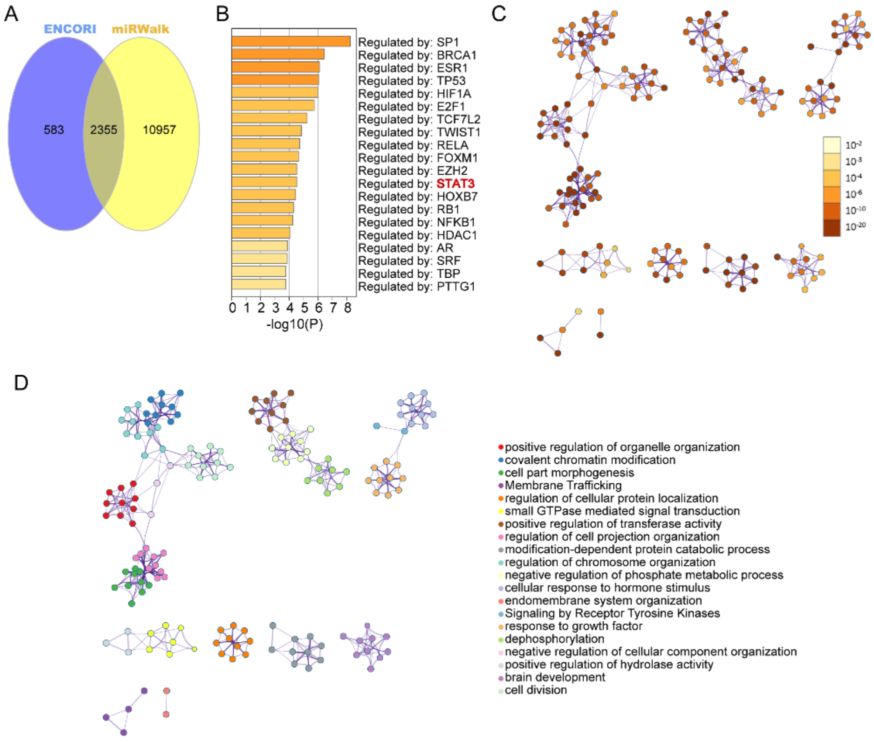

A. Bioinformatics analysis was used to predict the downstream regulatory genes of miR-34/449-5p, and the intersection analysis of Venn plot was drawn, and 2355 intersection genes were obtained. B. The signal pathway prediction analysis of 2355 genes were carried out to obtain the possible target protein data. C-D. The signal pathway prediction analysis of 2355 genes were carried out to obtain the possible

signal pathway data.

Supplementary Figure 3

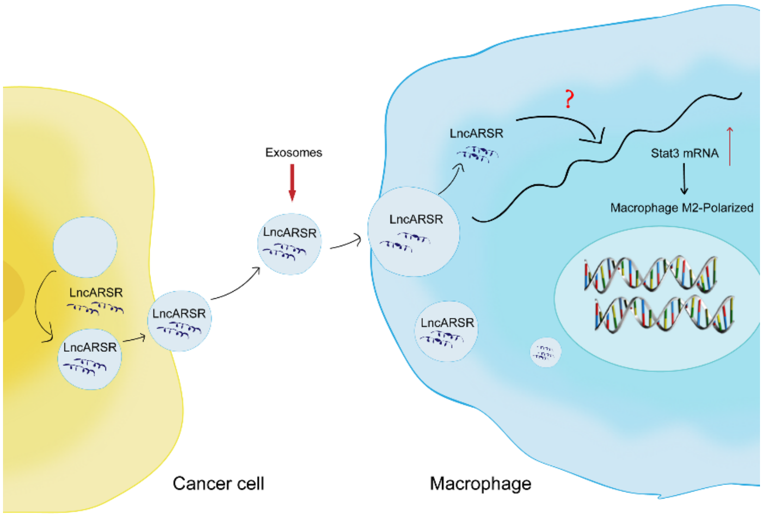

Tumor released exosomes can be taken up by macrophages and transmit biological information to macrophages, thus inducing the formation of TAM.
